# Adsorption of Serum Fetuin onto Octacalcium Phosphate and Its Relation to Osteogenic Property

**DOI:** 10.3390/ijms26031391

**Published:** 2025-02-06

**Authors:** Yuki Tsuboi, Ryo Hamai, Kyosuke Okuyama, Kaori Tsuchiya, Yukari Shiwaku, Kensuke Yamauchi, Osamu Suzuki

**Affiliations:** 1 Division of Craniofacial Function Engineering (Division of Biomaterials Science and Engineering), Tohoku University Graduate School of Dentistry, Sendai 980-8575, Japan; yuki.tsuboi.a8@tohoku.ac.jp (Y.T.); ryo.hamai.a3@tohoku.ac.jp (R.H.); kaori.tsuchiya.b6@tohoku.ac.jp (K.T.); yukari.shiwaku.a8@tohoku.ac.jp (Y.S.); 2 Division of Oral and Maxillofacial Reconstructive Surgery, Tohoku University Graduate School of Dentistry, Sendai 980-8575, Japan; okuyamakyousuke@icloud.com (K.O.); kensuke.yamauchi.a1@tohoku.ac.jp (K.Y.)

**Keywords:** octacalcium phosphate, fetuin, adsorption, osteoblasts

## Abstract

This study aimed to investigate how the chemical elements in relation to octacalcium phosphate (OCP) hydrolysis affect the osteoblastic differentiation in the presence of serum fetuin. The adsorption of fetuin onto OCP was examined in buffers having different degrees of supersaturation (DS) with respect to OCP and hydroxyapatite (HA) at pH 7.4 and 37 °C. The osteoblastic differentiation of mesenchymal stem cells (MSCs) was evaluated in cultures with OCP and 0 to 0.8 mg/mL of fetuin. The amount of fetuin adsorbed increased with increasing DS in the buffer. In the MSC culture, the coexistence of OCP and 0.2–0.4 mg/mL of fetuin close to serum level increased alkaline phosphatase activity; however, the activity was suppressed by 0.2–0.8 mg/mL of fetuin. Transmission electron microscopy revealed de novo crystal formation on OCP in supersaturated buffer and culture media with respect to OCP and HA at lower fetuin concentrations. Infrared spectroscopy and DS estimation indicate that the hydrolysis of OCP with de novo apatite formation was promoted in the culture media at 0.2–0.4 mg/mL of fetuin. These results suggest that OCP may promote osteoblastic differentiation if the suitable conditions are attained regarding the chemical elements and fetuin adsorption around OCP.

## 1. Introduction

Hydroxyapatite (HA), considered to be the prototype of the inorganic component of bone apatite crystal [1,2,3,4], belongs to a class of calcium phosphate materials, which exhibit high biocompatibility with bone tissue and have wide clinical application as bone substitute materials to fill bone defects [5]. Sintered HA is a non-resorbable material [5,6,7,8], whereas β-tricalcium phosphate (β-TCP) [9] and octacalcium phosphate (OCP) [10,11,12] are both classified as resorbable materials. The structure of OCP consists of an apatite layer stacked with a hydrated layer alternatively [1,13,14,15]. OCP is a metastable phase under physiological environment that tends to convert to HA [10,16,17,18,19,20,21,22,23]. OCP has recently been used clinically in dentistry in the form of a composite with collagen [24] and has begun to be used in the field of orthopedics in the form of a composite with gelatin [25]. When these calcium phosphate materials are implanted into bone defects, some proteins derived from the serum or synthesized by cells accumulate around these materials [20,26]. The interaction between the proteins and materials is considered to lead to biological responses such as immune reactions [27].

Osteocalcin (OC), which is a non-collagenous protein synthesized by osteoblasts and is abundant in serum, has been found to accompany OCP in locations where subsequent HA crystal formation occurs during human and rat bone mineralization [28]. The molecular structure of OC can be structurally matched and adsorbed onto the HA surface [29]. When the granule of synthetic OCP was implanted into a rat tibia bone defect, while another non-collagenous protein osteopontin (OP) was found within the newly formed bone matrix around the granules, the OC was present in accordance with the position of the OCP granule itself [30]. OC has been suggested to play a role in the migration of osteoclasts on the surface of β-TCP [31]. On this basis, it is reasonable to assume that the performance of OCP is controlled through interactions with biological proteins during bone regeneration [32].

Biochemical analysis and proteome analysis have shown that α2-HS glycoprotein (fetuin) can be accumulated in OCP [20,26]. The proteome analysis revealed that fetuin is involved in proteins commonly adsorbed onto OCP and HA, suggesting that fetuin may play a role in bone formation in conjunction with these calcium phosphate materials [33].

Fetuin is a serum-derived protein synthesized in the liver [34,35] and is known to be associated with the bone, wherein it can bind calcium ions [34,36] and form calcium and phosphate ion mineral complexes [37,38,39]. Fetuin inhibits apatite crystal growth outside of the collagen fibrils in vitro [37,38]. Fetuin has also been shown to regulate the proliferation of fibroblast-like cells [40,41] and control the differentiation of osteoblastic cells through its binding affinity with cytokines or by acting as an endogenous cytokine antagonist [42,43]. Thus, fetuin regulates bone mineralization and hard tissue formation. However, the role of fetuin in OCP-induced bone regeneration when it accumulates around OCP remains unelucidated.

OCP exhibits enhanced surface adsorption of bovine serum albumin (BSA), a serum-derived protein, under physiological conditions in vitro; this can be explained using the Langmuir-type adsorption model [44]. The adsorption affinity of BSA increases depending on the progress of the hydrolysis of OCP toward Ca-deficient HA (CDHA), a non-stoichiometric HA prepared in the presence of F^−^ ions at a low concentration of approximately 2 ppm [44]. Among the biologically related molecules, those that have the ability to interact with OCP include phosphorylated proteins [45,46], amelogenin (an enamel protein) [47], gelatin [48,49], collagen [50,51], and BSA [51]. The crystal-face-specific hydrophobic interactions of OCP have also been suggested to occur during amelogenin adsorption [47]. Thus, OCP has been shown to have a high affinity for biological proteins and behaves as a good adsorbent material. Elucidating the mechanism by which fetuin adsorption onto OCP affects OCP-enhanced bone formation is of notable interest.

The degree of supersaturation (DS) of the solution is the driving force for calcium phosphate precipitation [17,52]. Human serum has been reported to be almost saturated with respect to OCP [53], and the concentrations of serum Ca^2+^ and inorganic phosphate (Pi) ions are maintained constant through homeostasis [17]. Although the resorption by osteoclasts and formation by osteoblasts are known to repeat in the bone through homeostasis [54], the local inorganic ion concentration may fluctuate [55]. As OCP is a metastable phase under physiological conditions [16], Ca^2+^ uptake and Pi ion release should occur irreversibly and continuously in vivo [21,22]; thus, OCP hydrolysis affects its surrounding environment [12]. The solution supersaturation within a specific range enhances the adsorption of BSA onto OCP [56], thereby affecting the serum protein adsorption and related bone formation by OCP. However, although information on the adsorption of serum proteins to OCP and its related calcium phosphate materials is accumulating [12,57,58], it is unclear how adsorbed proteins modulate the properties of calcium phosphate materials and control the materials’ cellular responses. We hypothesized that fetuin, which accumulates on the surface of OCP materials in vivo [20,26], may be involved in regulating the material chemical properties of OCP and its related osteogenic ability through the adsorption. In this study, we investigated osteoblast differentiation related to OCP-enhanced bone formation from the viewpoint of the interaction between adsorbed fetuin and OCP.

## 2. Results

### 2.1. Adsorption of Fetuin onto OCP

#### 2.1.1. Adsorption Isotherms of Fetuin onto OCP Under Different Degrees of Supersaturations with Respect to OCP and HA

**Figure 1:**
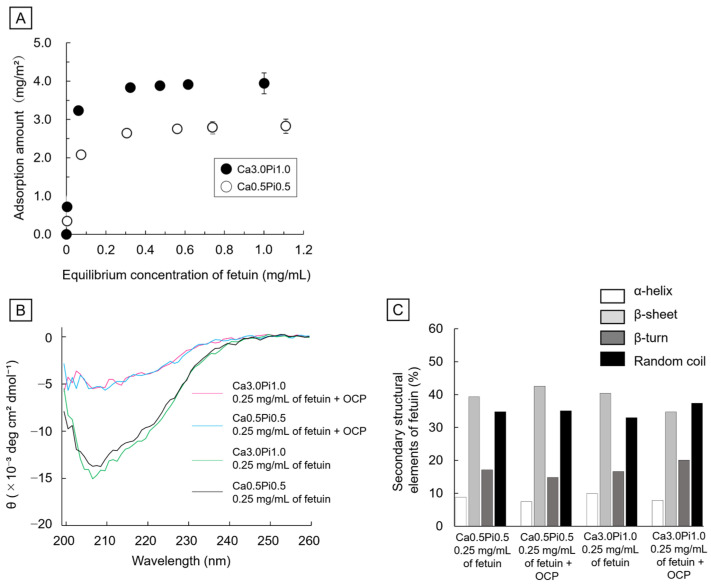
(**A**) Adsorption isotherms of fetuin onto OCP plotted as adsorption amount per unit surface area of OCP as a function of fetuin equilibrium concentration in the Tris-HCl buffers––0.5 mM Ca^2+^ with 0.5 mM Pi ions (Ca0.5Pi0.5) and 3.0 mM Ca^2+^ with 1.0 mM Pi ions (Ca3.0Pi1.0) at 37 °C. (**B**) CD spectra. (**C**) Secondary structural elements of fetuin in the Ca0.5Pi0.5 and Ca3.0Pi1.0 buffers in the presence of OCP with 0.25 mg/mL of fetuin.

Figure 1A shows the adsorption isotherms of fetuin onto OCP in 150 mM tris(hydroxymethyl)aminomethane-HCl (Tris-HCl) buffers containing 0.5 mM Ca^2+^ and 0.5 mM Pi ion (Ca0.5Pi0.5) or 3.0 mM Ca^2+^ and 1.0 mM Pi ion (Ca3.0Pi1.0) at pH 7.4 and 37 °C. The amount of fetuin increased in the equilibrium concentration range from 0 to approximately 0.3 mg/mL in both Tris-HCl buffers. Fetuin adsorption tended to saturate for equilibrium concentrations of over 0.3 mg/mL in the case of both isotherms. The adsorption amount of fetuin onto OCP incubated in Ca3.0Pi1.0 was larger than that in Ca0.5Pi0.5, regardless of the equilibrium concentrations of fetuin. The parameters for the adsorption of fetuin onto OCP were calculated using the approximations of isotherms based on the Langmuir model. The correlation coefficients were more than 0.99 for both isotherms. Adsorption equilibrium constants for Ca0.5Pi0.5 and Ca3.0Pi1.0 buffers were 33.5 and 69.4 mL/mg, respectively. The saturated adsorption amounts for the Ca0.5Pi0.5 and Ca3.0Pi1.0 buffers were 2.9 and 4.0 mg/m^2^, respectively. The equilibrium constants and saturated adsorption amounts were higher for the Ca3.0Pi1.0 buffer than for the Ca0.5Pi0.5 buffer.

#### 2.1.2. Circular Dichroism (CD) Spectra of Fetuin

Figure 1B shows the CD spectra of fetuin in the Ca0.5Pi0.5 and Ca3.0Pi1.0 buffers before and after incubation using OCP with 0.25 mg/mL of fetuin. The mean residual ellipticity decreased with decreasing fetuin concentration owing to the adsorption onto the OCP. The secondary structural elements of fetuin in Ca3.0Pi1.0 and Ca0.5Pi0.5 buffers were similar before the incubations with OCP granules (Figure 1C). Secondary structural elements of fetuin were retained after the incubation with OCP in each buffer.

#### 2.1.3. Fourier Transform Infrared (FTIR) Spectroscopic Analysis, Transmission Electron Microscope (TEM) Observation, and Selected Area Electron Diffraction (SAED) Analysis of OCP Before and After Fetuin Adsorption

**Figure 2:**
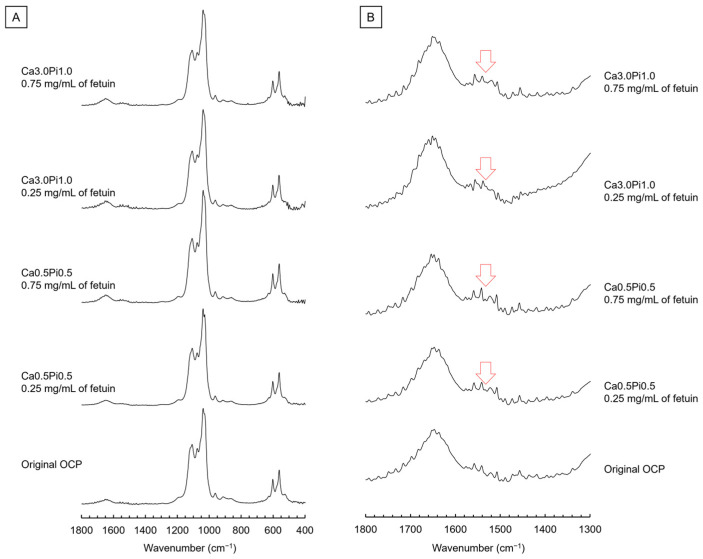
(**A**) FTIR spectra of OCP over the range of 400–1800 cm^−1^ and (**B**) 1300–1800 cm^−1^ before and after the incubations in the Ca0.5Pi0.5 and Ca3.0Pi1.0 buffers containing 0.25 and 0.75 mg/mL of fetuin. Arrows indicate amide II derived from fetuin.

Figure 2 shows the FTIR spectra of OCP before (original) and after the incubations in Ca0.5Pi0.5 and Ca3.0Pi1.0 buffers containing fetuin. In the spectra of the original OCP at 525 and 559 cm^−1^, the bands corresponding to *ν*_4_ HPO_4_ and *ν*_4_ PO_4_ were detected (Figure 2A). The bands of *ν*_3_ PO_4_ and *ν*_3_ PO_4_/HPO_4_ appeared in these spectra at 1023 and 1075 cm^−1^, respectively. The bands corresponding to *ν*_3_ HPO_4_(5) and *ν*_3_ HPO_4_(6) were also observed at 1105 and 1120 cm^−1^, respectively. After incubation in Ca0.5Pi0.5, and Ca3.0Pi1.0 buffers, the appearance of the bands corresponding to PO_4_ and HPO_4_ was maintained regardless of the fetuin concentration. The bands corresponding to H_2_O were also detected in the spectra of the original at 1644 cm^−1^ (Figure 2B). However, the new band appeared in the spectra of OCP incubated in both buffers containing fetuin at 1537 cm^−1^, and this could be identified as amide II.

**Figure 3:**
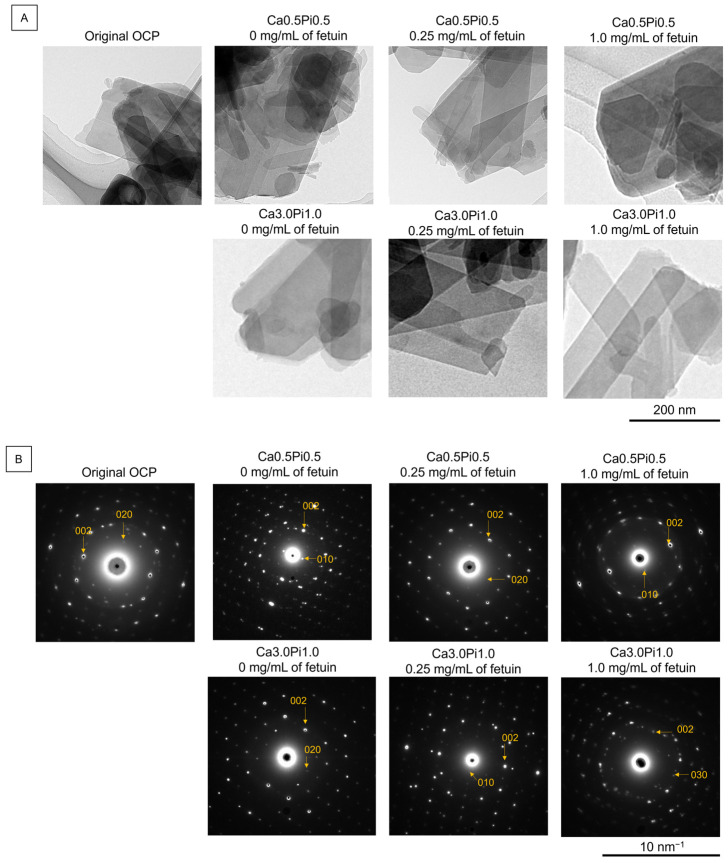
(**A**) Lower magnified TEM images and (**B**) SAED patterns of OCP before (original) and after the incubations in the Ca3.0Pi1.0 and Ca0.5Pi0.5 Tris-HCl buffers with 0, 0.25, and 1.0 mg/mL of fetuin. The SAED patterns indicate the reflections along the [100] zone axis of OCP. Bars in the TEM images and SAED patterns represent 200 nm and 10 nm^–1^, respectively.

Figure 3 shows the TEM images and SAED patterns of the OCP before (original) and after treatment with Tris-HCl buffers containing fetuin at different concentrations of Ca^2+^ and Pi. Plate-like particles, which are typical morphologies of OCP crystals, are observed in the TEM image of the original OCP. The diffraction spots corresponding to 020 and 002 of OCP were detected in the SAED pattern of the original OCP at 4.58 and 3.44 Å, respectively, indicating that the plate-like particles were single crystals of OCP. The diffraction spots corresponding to 002 were aligned along the long-axis direction of the plate-like particles. Thus, the plate-like OCP crystals grew well along the *c*-axis. After incubation in the Tris-HCl buffers, regardless of the Ca^2+^, Pi, and fetuin concentrations, the particles maintained a plate-like morphology. Diffractions corresponding to 020 and 002 were also detected in the SAED patterns.

**Figure 4:**
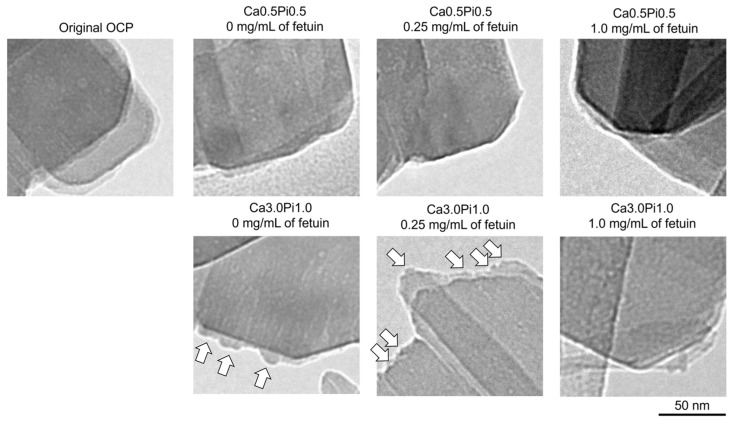
Higher magnified TEM images of OCP before (original) and after the incubations in the Ca3.0Pi1.0 and Ca0.5Pi0.5 Tris-HCl buffers with 0, 0.25, and 1.0 mg/mL of fetuin. Bars in the TEM images represent 50 nm. Open arrows indicate the de novo depositions formed on the edge of plate-like OCP particles.

Figure 4 shows the magnified TEM images of OCP before and after incubation in the Tris-HCl buffers. The edge of the plate-like particle was smooth in the TEM image of OCP before and after the incubations in the Ca0.5Pi0.5 buffer containing 0.25 and 1.0 mg/mL of fetuin. However, the formation of small protrusions was observed on the edges of the plate-like particles after incubation in the Ca3.0Pi1.0 buffer at 0 and 0.25 mg/mL of fetuin. The number of small protrusions tended to be larger on the OCP incubated at 0.25 mg/mL of fetuin compared to 0 mg/mL of fetuin. After incubation in the Ca3.0Pi1.0 buffer with 1.0 mg/mL of fetuin, no small protrusions were observed at the edge of the plate-like OCP.

### 2.2. Cell Culture Experiments

#### 2.2.1. DNA Concentrations and Alkaline Phosphatase (ALP) Activities

**Figure 5:**
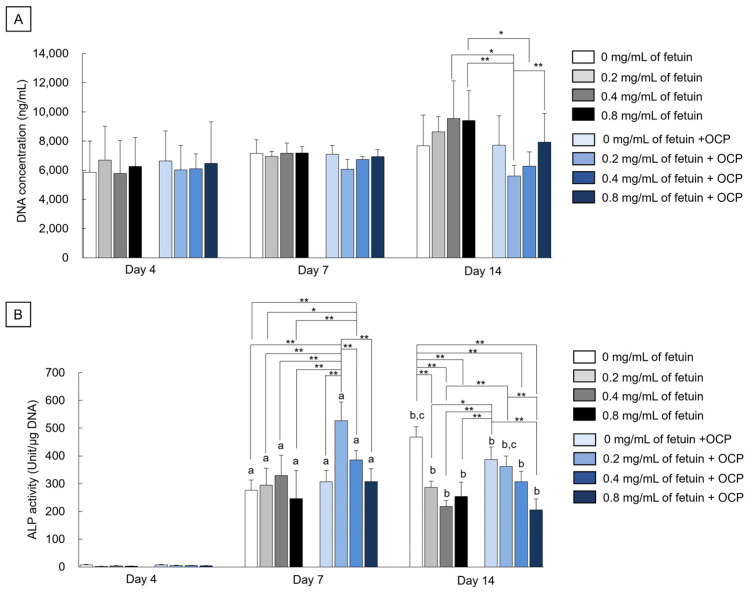
(**A**) DNA concentrations and (**B**) ALP activities of D1 cells in the presence and absence of OCP granules at 0, 0.2, 0.4, and 0.8 mg/mL of fetuin after the cultivations for 4, 7, and 14 days. ** *p* < 0.01, * *p* < 0.05. ^a^ *p* < 0.05 indicates a significant difference between day 4 and day 7. ^b^ *p* < 0.05 indicates a significant difference between day 4 and day 14. ^c^ *p* < 0.05 indicates a significant difference between day 7 and day 14.

Figure 5A shows the DNA concentrations in mouse bone marrow-derived mesenchymal stem cell (MSC) line D1 cells after incubation in the presence of fetuin and OCP on days 4, 7, and 14. The DNA concentrations tended to increase in the 0.2, 0.4, and 0.8 mg/mL of fetuin groups from day 7 to day 14. However, the DNA concentrations were maintained in the 0 mg/mL of fetuin group and 0, 0.2, 0.4, and 0.8 mg/mL of fetuin + OCP groups during the incubations for 14 days. On days 7 and 14, the DNA concentration decreased with decreasing fetuin concentration in the range of 0.2–0.8 mg/mL in the presence of OCP granules, although the DNA concentrations tended to be lower in the 0.2 mg/mL of fetuin + OCP and the 0.4 mg/mL of fetuin + OCP groups than in the 0 mg/mL of fetuin + OCP and 0–0.8 mg/mL of fetuin groups. A significant difference was observed among the 0.2 mg/mL of fetuin + OCP and 0.8 mg/mL of fetuin + OCP groups, the 0.4 mg/mL of fetuin group, and the 0.8 mg/mL of fetuin group on day 14. In addition, the concentration significantly decreased in the 0.4 mg/mL of fetuin + OCP group as compared to the 0.8 mg/mL of fetuin group on day 14.

Figure 5B shows the alkaline phosphatase (ALP) activity of the D1 cells normalized to DNA concentrations on days 4, 7, and 14. Normalized ALP activity increased in all groups from days 4 to 7. Although ALP activities in the 0–0.8 mg/mL of fetuin groups and 0 mg/mL of fetuin + OCP group were similar values on day 7, the ALP activities increased with decreasing fetuin concentration in the range of 0.2–0.8 mg/mL in the presence of OCP. The ALP activity for the 0.2 mg/mL of fetuin + OCP group was the highest among all groups on day 7, and a significant difference was observed between the 0.2 mg/mL of fetuin + OCP and other groups. The ALP activities in the 0.2–0.8 mg/mL of fetuin groups were maintained from days 7 to 14, although the activity increased in the 0 mg/mL of fetuin group. ALP activity also increased in the 0 mg/mL of fetuin + OCP group on day 14 compared to that on day 7. However, the ALP activities for 0.2–0.8 mg/mL of fetuin + OCP groups at day 14 were lower than those on day 7. The trend of ALP activity was similar in the 0.2–0.8 mg/mL of fetuin + OCP group between days 14 and 7. Significant differences were observed among the 0.2 mg/mL of fetuin + OCP group and the 0.8 mg/mL of fetuin + OCP or 0.4 mg/mL of fetuin group. On day 14, the ALP activity was significantly higher in the 0 mg/mL of fetuin group than in the 0.2–0.8 mg/mL of fetuin groups and 0.8 mg/mL of fetuin + OCP groups. In addition, the ALP activity significantly increased in the 0 mg/mL of fetuin + OCP group compared to the 0.2–0.8 mg/mL of fetuin groups and the 0.4–0.8 mg/mL fetuin group + OCP groups at day 14. These results were confirmed by the statistical analysis indicated by the difference in significance.

#### 2.2.2. TEM Observation and FTIR Analysis of OCP Incubated with MSCs and Fetuin

**Figure 6:**
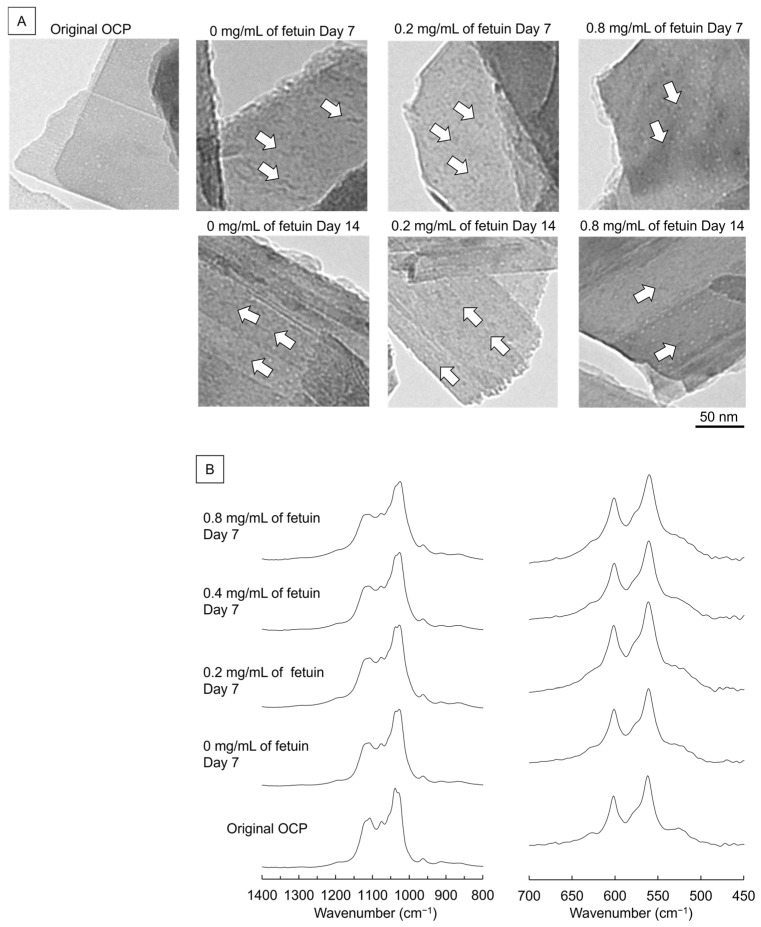
(**A**) TEM images and (**B**) FTIR spectra of OCP before and after the cultivations of D1 cells in the presence of fetuin at day 7 (**A**,**B**) and day 14 (**A**). Bars in the TEM images represent 50 nm. Open arrows indicate the de novo depositions formed on the *a*-plane of OCP crystals.

Figure 6A shows the TEM images of OCP before and after incubation with MSCs and fetuin on days 7 and 14, respectively. The plate-like morphology was maintained after incubation for 14 days, regardless of fetuin concentration. Fine de novo depositions were formed on the surface of the plate-like particles of OCP after the incubation in the presence of 0 and 0.2 mg/mL of fetuin at day 7. However, the amount of de novo deposition was apparently smaller on the surface of plate-like particles after the incubations in the presence of 0.8 mg/mL of fetuin compared to 0 and 0.2 mg/mL of fetuin at day 7. Although the amount of fine depositions on the surface in the presence of 0 and 0.2 mg/mL of fetuin tended to increase with the incubation periods of 14 days, the plate-like particles incubated with 0.8 mg/mL of fetuin maintained the surface with a smaller amount of deposition.

Figure 6B shows the FTIR spectra of OCP after the cultivation of MSCs in the presence of fetuin for seven days. The intensities of bands corresponding to *ν*_3_ HPO_4_(5) at 1103 cm^−1^ and *ν*_3_ HPO_4_(6) at 1121 cm^−1^ slightly decreased in the spectra of incubated OCP regardless of fetuin concentrations. The bands attributed to *ν*_4_ HPO_4_ at 525 cm^−1^ also decreased after the incubations. However, the intensities of these *ν*_3_ HPO_4_ bands tended to be lower in the spectra of OCP with 0.2 mg/mL of fetuin than in the spectra of OCP with 0, 0.4, and 0.8 mg/mL of fetuin. In fact, it was apparent that the comparative intensities between *ν*_3_ HPO_4_(5) at 1103 cm^−1^ or *ν*_3_ HPO_4_ at 1121 cm^−1^ of OCP and *ν*_3_ PO_4_/HPO_4_ at 1075 cm^−1^ were slightly lower in OCP with 0.2 mg/mL of fetuin than in the original OCP or OCP with 0.4 and 0.8 mg/mL of fetuin.

#### 2.2.3. Ion Concentration and DS Changes in the Culture Media

Table 1 shows the changes in ion composition and DS with respect to calcium phosphates before and after cultivation of D1 cells with and without fetuin. The Ca^2+^ concentration decreased, and the Pi ion concentration increased with the incubation period in all groups. However, Ca^2+^ concentrations in the 0.2 and 0.4 mg/mL of fetuin + OCP groups were lower than in the 0.2–0.8 mg/mL of fetuin, 0 mg/mL of fetuin + OCP, and 0.8 mg/mL of fetuin + OCP groups on day 7. Although Pi ion concentrations increased in the 0–0.8 mg/mL of fetuin + OCP groups compared to the 0–0.8 mg/mL of fetuin groups on day 7, the Pi ions tended to be lower in the 0.2 and 0.4 mg/mL of fetuin + OCP groups than in the 0 and 0.8 mg/mL of fetuin + OCP groups. Pi ions also decreased with decreasing fetuin concentration in the absence of OCP. The Ca^2+^ concentrations in the 0–0.8 mg/mL of fetuin + OCP groups showed similar tendencies between days 7 and 14. After the incubations for 14 days, the Pi ion in the 0.2 mg/mL of fetuin + OCP group was the lowest in the fetuin + OCP groups.

Table 1 also shows the values of DS with respect to HA, OCP, and DCPD calculated using the measurement results of Ca^2+^ and Pi ion concentrations and pH values at 37 °C. The DS value indicated that the original culture medium was supersaturated with respect to HA and OCP and saturated with respect to DCPD. The DS of HA was higher than that of OCP in the media before and after incubation. Although the incubated media were also supersaturated with respect to HA and OCP, the DS values with respect to HA and OCP decreased with the incubation period in all groups. The DS values were lower in the media incubated with OCP and fetuin than in the media incubated with fetuin alone. In addition, the DS with respect to HA and OCP tended to decrease in the media of the 0.2 and 0.4 mg/mL of fetuin + OCP compared to the 0 and 0.8 mg/mL of fetuin + OCP at day 7 and day 14.

## 3. Discussion

The present study focused on the fetuin, a serum protein, which is known to be present ubiquitously in vivo and was proved to be accumulated on OCP implanted in bone and subcutaneous tissues [26]. OCP tends to convert to the most stable phase HA (CDHA) and enhances bone formation and osteoblastic differentiation more than CDHA or HA [22,59]. To elucidate the interaction and the effect of the fetuin, the fetuin adsorption, OCP hydrolysis to CDHA and their relation to osteoblastic differentiation were analyzed and discussed.

The results of this study indicate that the hydrolysis of OCP with de novo apatite formation and adsorption of fetuin onto OCP may cooperatively promote the osteoblastic differentiation of MSCs under supersaturation with respect to HA and OCP at 0.2–0.4 mg/mL of fetuin close to the serum level [60]. The adsorption amount increased with increasing DS for OCP and HA in the isotherms of fetuin, as approximated by the Langmuir model (Figure 1). The D1 cells showed higher ALP activities in the presence of OCP and 0.2–0.4 mg/mL of fetuin compared to OCP without fetuin, although the fetuin without OCP suppressed increasing ALP activities (Figure 5). In the buffer of Ca3.0Pi1.0 and in cell culture media, de novo crystals formed on the OCP crystals at lower concentrations of fetuin (Figure 4 and Figure 6A). Interestingly, the hydrolysis of OCP was promoted in the culture media with fetuin over 0.2–0.4 mg/mL (Figure 6B and Table 1). Thus, the adsorption of fetuin onto OCP under higher DS conditions could contribute to reducing the inhibitory effect of fetuin on osteoblastic activity [41], but also to the higher reaction rate of OCP hydrolysis, changing the Ca^2+^ and Pi concentrations [32], resulting in the promotion of the osteoblastic differentiation of MSCs depending on the fetuin concentration. Since OCP is a metastable phase under physiological conditions [16], it tends to spontaneously and irreversibly hydrolyze to CDHA accompanied by Ca^2+^ and Pi ion exchange [16,17,21] and serum protein adsorption [20,26,33,44,56]. OCP enhances osteoblast differentiation [22,61] and osteocyte differentiation [12] from osteoblast precursor cells while its hydrolysis progresses [12]. OCP also enhances macrophage migration [12] and the formation of osteoclasts from the co-culturing of osteoblasts and bone marrow macrophages without adding osteoclast differentiation factors, with an increasing expression of the osteoclast differentiation factor in osteoblasts during its hydrolysis process [12]. Moreover, a study revealed that the introduction of lattice defects into the OCP structure increases its apparent solubility and the resultant hydrolysis rate, which further enhances the osteoblastic differentiation of MSCs and new bone formation [32,48].

Adsorption analyses revealed that the amount of fetuin adsorbed onto the OCP increased with increasing DS with respect to OCP and HA (Figure 1A). The FTIR spectra of amide II indicated that the adsorption of fetuin onto OCP occurred in the buffers (Figure 2). The adsorption isotherms approximated by the Langmuir model indicated that the adsorption affinity of fetuin for OCP and the number of adsorption sites increased with increasing DS values (Figure 1A). Protein adsorption onto calcium phosphate is regulated by changes in the secondary structure of the protein [62]. However, CD spectra showed no changes in the conformation of fetuin in the buffers, regardless of DS values. (Figure 1B,C). The surface charge regulated by Ca^2+^ and Pi ion adsorption and the chemical compositions affect the adsorption of BSA onto HA [63,64]. X-ray photoelectron spectroscopy revealed that the molar ratio of HPO_4_^2−^/PO_4_^3−^ on the surface of OCP increased in simulated body fluid (SBF) compared to that in Tris-HCl without Ca^2+^ and Pi [48]. In this study, TEM observations and SAED analyses indicated that de novo crystals of OCP grew toward the *c*-axis and formed on the edge of the plate-like OCP particles at a lower concentration of fetuin (Figure 3 and Figure 4). The formation of de novo crystals on the edges of OCP crystals is associated with the additional formation of adsorption sites for BSA when the DS with respect to OCP and HA increases [56]. Therefore, changes in the chemical composition of the surface and de novo crystal formation depends on the DS values, which could regulate the adsorption affinity and adsorption sites of fetuin under physiological conditions.

Osteoblast differentiation begins with the expression of ALP, followed by the expression of collagen, osteopontin, and osteocalcin [65]. In the process of osteoblast differentiation, transcription factors, such as osterix and Runx2, are involved in regulating their expression [66]. The previous study has shown that ALP expression of MSCs induced by OCP decreases over time after reaching its peak, suggesting that ALP expression serves as an indicator of the transition of early differentiation [67]. The previous study also observed that osteocalcin gene expression is detected in MSC spheroid culture with OCP by expressing osteocalcin [68]. Further study confirmed that osteocalcin secretion by MSC is detected in a culture study together with OCP through the analysis of ELISA while ALP is expressed in the early stage of the differentiation of MSC [69]. In the culture of MSCs, ALP activity, a marker of osteoblastic differentiation at an early stage, of D1 cells decreased in the presence of 0.2–0.8 mg/mL of fetuin compared with 0 mg/mL of fetuin, although the DNA concentrations of cells increased with incubation periods in the presence of fetuin (Figure 5). These results show similar tendencies regarding the positive and negative effects of fetuin on cell proliferation [40] and osteoblastic differentiation [41], respectively, as reported in the literature. The fetuin acts as an antagonist of TGF-β and BMP produced from the cells [41].

The ALP activities of cells increased with decreasing the concentration in the range from 0.2 to 0.8 mg/mL of fetuin in the presence of OCP (Figure 5B). This suggests that the adsorption of fetuin onto OCP may reduce the negative effects of fetuin [41]. However, new fetuin was added during the cell culture experiments every two days in this study. De novo depositions were also observed on the OCP in the culture media, as well as in the Ca3.0 Pi1.0 buffer, and these amounts apparently increased with incubation periods (Figure 6A) at 0 and 0.2 mg/mL of fetuin. The culture medium maintained supersaturation with respect to OCP and HA during cultivation (Table 1). These results suggest that fetuin can be continuously adsorbed through the creation of new adsorption sites in OCP for serum proteins [56] to maintain a lower concentration of fetuin during cultivation.

However, the osteoblastic differentiation of MSCs promoted in the presence of OCP and 0.2–0.4 mg/L of fetuin than in the presence of OCP without fetuin (Figure 5B). To elucidate this mechanism, we focused on the hydrolysis of OCP in the presence of fetuin. A higher rate of OCP hydrolysis is associated with enhanced osteoblastic activity [32]. According to the zone axis of diffraction of the original OCP (Figure 3B), de novo depositions formed on the *a*-plane of OCP after the incubations with MSCs at 0 and 0.2 mg/mL of fetuin (Figure 6A). The morphology of de novo deposition is similar to the formation of apatite on the *a*-plane of OCP in vivo and in vitro [48,70]. The culture medium was supersaturated with respect to HA (Table 1). These results indicate that apatite or its precursors may form on the *a*-plane of the OCP after incubation with lower concentrations of fetuin. The decrease in the infrared adsorption bands of HPO_4_^2−^ in the OCP structure and the changes in Ca^2+^ and Pi concentrations in the culture media also indicated that the hydrolysis of OCP progressed after cell culture in the presence of fetuin. However, the lower concentration of Ca^2+^ in the media and the decreasing HPO_4_^2−^ in OCP structure (Figure 6B and Table 1) suggest that the OCP hydrolysis could be promoted at 0.2–0.4 mg/mL of fetuin. The smaller amount of deposition on OCP could correspond to the suppression of OCP hydrolysis at 0.8 mg/mL of fetuin (Figure 6A).

Acidic serum proteins such as BSA and fetuin control the formation of de novo calcium phosphates under physiological conditions. Fetuin inhibited the precipitation of calcium phosphate in solution at a concentration of 5 mg/mL [37]. However, fetuin contributes to the stabilization of amorphous calcium phosphate clusters [71,72]. A large amount of BSA adsorption inhibits the de novo formation of OCP on collagen and plate-like OCP crystals [51,56,73]. The adsorption of BSA also tends to suppress the hydrolysis reaction of OCP in the presence of F^−^ [44]. In contrast, lower concentrations of BSA promote de novo OCP deposition on collagen and OCP crystals [51,56,73]. Combes et al. proposed that a lower adsorption amount of BSA enhances the nucleation rate via the stabilization of the nucleus, and its suppressive effect on crystal growth is limited [73]. Ito et al. reported that the formation of apatite nuclei is an important process in the transformation of OCP into HA [74]. In contrast, a larger amount of adsorbed BSA inhibits the supply of ions for crystal growth [73]. The adsorption equilibrium constants were higher in fetuin (2114 mL/μmol) (Figure 1A) than in BSA (1520 mL/μmol) [44] regarding the adsorption onto OCP in the buffer of Ca0.5Pi0.5, suggesting that the capacity to stabilize the nucleation could be higher in fetuin than in albumin. In addition, the adsorption equilibrium constant for fetuin approximately doubled with increasing DS (Figure 1A). As indicated by these previous studies, the crystal growth of de novo deposition on the OCP (Figure 4 and Figure 6A) could be inhibited by the larger adsorption amount of fetuin in the buffer and cell culture environment. In contrast, the adsorption of a lower amount of fetuin could assist the formation of nucleus of apatite or its precursor on the surface of OCP and facilitate crystal growth, resulting in the hydrolysis of OCP promoted in the cell culture at lower fetuin concentrations (Figure 5B and Figure 6, and Table 1). Another possibility is that at a concentration around 0.2 mg/mL of fetuin, the adsorption amount at the equilibrium concentration of the adsorption isotherm is in the concentration region toward adsorption saturation (Figure 1). When the concentration is lower than the adsorption saturation, the unadsorbed sites of the molecules of fetuin per unit surface area may effectively promote the dissolution of the OCP surface, resulting in the acceleration of the surface hydrolysis. This could be a mechanism by which a part of the surface contributes to promoting osteoblast differentiation. The possibility that crystal growth is observed due to the unadsorbed sites of the molecules has been reported and discussed in the previous study of phosphoserine onto HA adsorption [75]. 

The accumulation of fetuin and de novo apatite formation occurred on the surface of the OCP implanted on the mouse calvarial bone [20,26,70]. The results of the adsorption analyses (Figure 1) and morphological observations (Figure 3, Figure 4 and Figure 6A) suggested that de novo appetite formation could contribute to the accumulation of fetuin on OCP in vivo. A higher DS with respect to HA under physiological conditions is the driving force for the hydrolysis of OCP with the formation of apatite [12]. Although serum is almost saturated and supersaturated with respect to OCP and HA [53], the self-dissolution of OCP and the osteoclastic resorption of OCP could increase the DS around the implanted OCP [32]. The mechanism of OCP transformation into HA via hydrolysis is a dissolution–reprecipitation process [16]. Interestingly, the coexistence of OCP and fetuin likely promoted the osteoblastic differentiation of MSCs and the hydrolysis reaction of OCP through the fetuin adsorption at 0.2–0.4 mg/mL of fetuin (Figure 5B and Figure 6, Table 1). The concentration of fetuin is approximately 0.4–0.6 mg/mL in serum [60]. De novo apatite formation on OCP allows for the continuous serum fetuin adsorption to reduce the inhibitory effect of fetuin on osteoblastic activity [41] under supersaturated conditions with respect to OCP and HA. However, the progress of OCP hydrolysis contributing to the activation of osteoblasts [32] could be dependent on serum fetuin concentration. Therefore, the findings of this study indicate that the hydrolysis of OCP accompanying de novo apatite formation and the accumulation of serum fetuin [20,26,70] could be involved in OCP-induced bone regeneration.

Although the precise osteoblastic differentiation processes enhanced by OCP with fetuin have not been completely clarified, the results of the present study suggest the possible mechanism as follows: (1) the rate of hydrolysis of OCP is regulated by fetuin through adsorption depending on its concentration around OCP; (2) de novo OCP crystal deposition onto the original OCP is taking place under a certain fetuin concentration (around 0.2 mg/mL); (3) the enhanced crystal deposition is associated with the enhancement of the hydrolysis of the original OCP hydrolysis; (4) MSC differentiation is enhanced by the intrinsic capacity of OCP through the advancement of the hydrolysis of OCP under the limited concentration of the fetuin-adjacent OCP surface. The limitations of the present study are considered as follows: (1) the adsorption property of fetuin in the presence of other serum proteins is still unclear; therefore, (2) whether the bilayer or multiple adsorptions of serum proteins affect the effect of fetuin is still unclarified on the osteoblastic differentiation of OCP; also, (3) the relevance of the present in vitro analysis needs to be confirmed through in vivo bone formation by OCP implantation. The present study, however, has clarified the role of fetuin under physiological conditions and its relationship with OCP, which is an osteoconductive bone substitute material. The present results may provide useful information for the use of OCP in clinical applications.

## 4. Materials and Methods

### 4.1. Preparation of OCP Granules

The granules of OCP material were prepared for the adsorption and cell culture experiments. OCP was prepared via the direct precipitation method by mixing a calcium acetate solution and a disodium hydrogen phosphate solution under supersaturated conditions with respect to HA and OCP. The OCP precipitate was then washed and dried at 105 °C for 24 h. The dried precipitate was ground and sieved to prepare the OCP granules with diameters of <53 μm as well as 300–500 μm for the adsorption and cell culture experiments, respectively. The OCP granules used for cell culture experiments were sterilized at 120 °C for 2 h.

### 4.2. Adsorption Experiments

**Figure 7:**
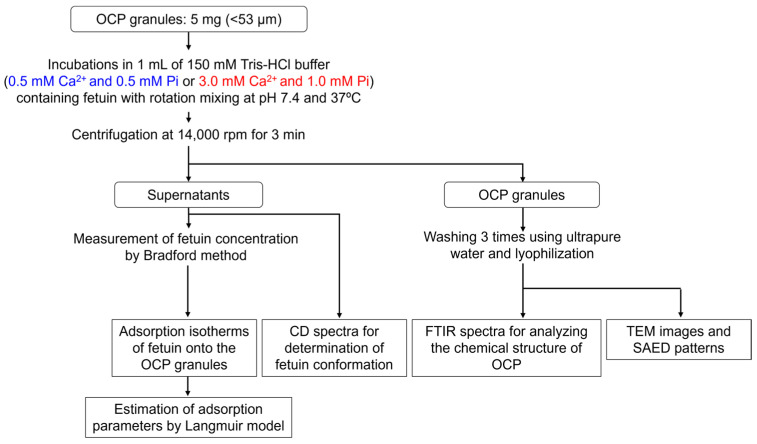
Flowchart of adsorption experiments. The experiments were designed to examine how the adsorption behavior of OCP is regulated by the surrounding chemical environment under physiological conditions. Adsorption isotherms were measured by the incubations of OCP granules (<53 μm) in the fetuin-containing Tris-HCl buffer saturated (0.5 mM Ca^2+^ and 0.5 mM Pi) and supersaturated (3.0 mM Ca^2+^ and 1.0 mM Pi) with respect to OCP. The adsorption isotherms were approximated using the Langmuir model to estimate the adsorption affinity of fetuin for OCP. The conformation changes in fetuin were examined in the physiological conditions in the presence of OCP by measurements of CD spectra. The structural and morphological changes in OCP during the fetuin adsorption were analyzed by FTIR and SAED. The morphological changes in OCP after the incubations were observed using TEM.

A flowchart of adsorption experiments is shown in Figure 7. The adsorption experiment was designed to simulate the physiological environment. Two Tris(hydroxymethyl)aminomethane-HCl (Tris-HCl) buffer solutions (150 mM)—containing 0.5 mM CaCl_2_·2H_2_O with 0.5 mM KH_2_PO_4_ and 3.0 mM CaCl_2_·2H_2_O with 1.0 mM KH_2_PO_4_—were prepared at pH 7.4 and 37 °C in accordance with the procedure followed in our previous studies [56]. The buffer containing 0.5 mM Ca^2+^ and 0.5 mM Pi (Ca0.5Pi0.5) was saturated (DS = 1.09 × 10^0^) with respect to OCP, while that containing 3.0 mM Ca^2+^ and 1.0 mM Pi (Ca3.0Pi1.0) was supersaturated (DS = 7.95 × 10^5^) with respect to OCP [56]. The DS values with respect to HA for the Ca0.5Pi0.5 and Ca3.0Pi1.0 were 9.97 × 10^7^ and 4.28 × 10^12^, respectively [56]. Fetuin from bovine serum (Mw = 48.4 kDa, Sigma-Aldrich, St. Louis, MO, USA) was dissolved in both buffers at 0, 0.10, 0.25, 0.50, 0.75, 1.0, and 1.5 mg/mL. OCP granules (5 mg) with diameters below 53 μm and with a specific surface area of 16 m^2^/g were soaked in 1 mL of these fetuin-containing solutions at pH 7.4 and 37 °C for 1 h via rotation mixing. After incubation, the supernatants were obtained using centrifugation at 14,000 rpm for 3 min. The concentrations of fetuin in the supernatants were measured using the Bradford method with Coomassie brilliant blue (CBB) protein assay solution (Nacalai Tesque Co., Ltd., Kyoto, Japan) to estimate the adsorption isotherms on the OCP granules. The fetuin concentration range to determine the adsorption isotherm can be measured by the protein assay in the present study. The total concentration of serum proteins, including fetuin, has previously been reported to be able to be estimated by the Bradford method [76]. The adsorption isotherms were approximated using the Langmuir adsorption isotherm model shown in Equation (1).

*Q* = (*K*⸱*Q*_0_⸱*C*)/(1 + *K*⸱*C*) (1)

where *Q* is the amount of adsorbate adsorbed, *C* is the equilibrium adsorbate concentration, *K* is the equilibrium constant, and *Q*_0_ is the saturation capacity of the adsorbate.

The conformations of fetuins before and after incubation in the presence of OCP granules in the Tris-HCl buffers of Ca0.5Pi0.5 and Ca3.0Pi1.0 were determined using a circular dichroism (CD) spectrometer (J-805, JASCO Corporation, Tokyo, Japan). The CD spectra were measured over a wavelength range of 200–260 nm in a quartz cell with a 1.0 cm path length and a response time of 1 s at a scan rate of 100 nm/min. The obtained CD spectra were analyzed using the software of the spectrometer system.

After the adsorption of fetuin, the OCP granules were washed three times using ultrapure water and lyophilized. The specimens were analyzed using Fourier transform infrared (FTIR) spectroscopy (FT/IR-6300, JASCO Corporation, Tokyo, Japan). The FTIR spectra of the specimens diluted by KBr were measured over the range of 1800–400 cm^−1^ with a resolution of 4 cm^−1^. The morphologies and selected area electron diffraction (SAED) patterns of the OCP were observed using field emission transmission electron microscopy (FE-TEM; JEM-2100F, JEOL Ltd., Tokyo, Japan) before and after incubation in fetuin-containing buffers

### 4.3. Cell Culture Experiments

#### 4.3.1. Analysis of Cell Growth and Osteoblastic Differentiation

**Figure 8:**
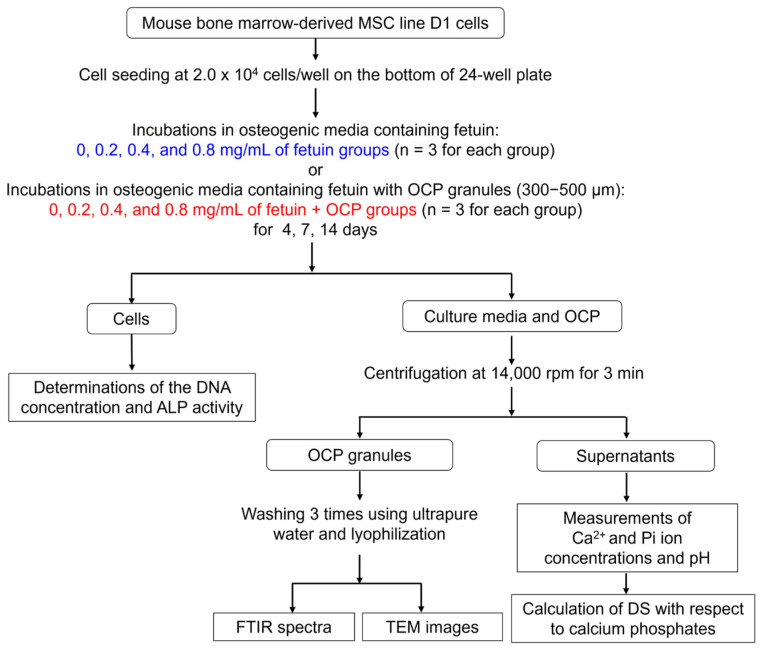
Flowchart of cell culture experiments. The experiments were designed to examine the relationship between OCP hydrolysis in the presence of fetuin and the osteoblastic differentiation of MSCs. D1 cells were cultured in the osteogenic media containing fetuin (0, 0.2, 0.4, and 0.8 mg/mL) in the absence of and presence of OCP granules (300–500 μm). At days 4, 7, and 14 of incubations, the DNA concentration and ALP activity of cells were determined to analyze the proliferation and osteoblastic differentiation of cells. The incubated OCP granules were characterized by FTIR to evaluate the progress of OCP hydrolysis in the presence of fetuin and cells. The formation of de novo crystals during the hydrolysis was observed using TEM. The concentrations of ions and pH in the supernatants of culture media were measured. The solubility of calcium phosphates in the media was estimated by calculating DS with respect to HA, OCP, and DCPD using the analytical values of ion concentrations and pH.

A flowchart of cell culture experiments is shown in Figure 8. The osteoblastic cell differentiation was examined utilizing the mesenchymal stem cell (MSC) line. Mouse bone marrow-derived MSC (D1 cells) purchased from ATCC (Rockville, MD, USA) were used for cell culture experiments. The osteogenic medium was prepared by adding 10% fetal bovine serum (ThermoFisher Scientific, Waltham, MA, USA), 1% penicillin–streptomycin solution (Nacalai Tesque, Inc., Kyoto, Japan), 50 μg/mL of ascorbate 2-phosphate (Sigma-Aldrich Co. LLC, St. Louis, MO, USA), 10 mM β-glycerophosphate (Tokyo Chemical Industry Co., Ltd., Tokyo, Japan), and 100 nM dexamethasone (Sigma-Aldrich Co. LLC) into Dulbecco’s Modified Eagle’s Medium (FUJIFILM Wako Pure Chemical Co., Ltd., Japan). Fetuin was dissolved in osteogenic medium at concentrations of 0, 0.2, 0.4, and 0.8 mg/mL.

D1 cells (passage 5) were seeded on the bottom of a 24-well plate at 2.0 × 10^4^ cells/well. Transwell inserts with 8.0 μm pores (FALCON^®^ Cell Culture Insert, Corning Inc., Corning, NY, USA) were applied into each well, and 4 mg of sterilized OCP granules with diameters within 300–500 μm were placed on the insert. The D1 cells were incubated in 1 mL of osteogenic media containing 0, 0.2, 0.4, and 0.8 mg/mL of fetuin in the presence of OCP granules (*n* = 3 for each group) in a 5% CO_2_ and 95% air atmosphere under humidified conditions at 37 °C for 4, 7, and 14 days. The D1 cells were also cultured in media containing 0, 0.2, 0.4, and 0.8 mg/mL of fetuin in the absence of OCP granules (*n* = 3 for each group). The osteogenic media with different concentrations of fetuin were changed every 2 days.

After the incubations at 4, 7, and 14 days, the D1 cells were lysed in 250 μL of 0.2% Triton X-100 solution (Sigma-Aldrich, St. Louis, MO, USA) by using a sonicator. The DNA concentrations and alkaline phosphatase (ALP) activities of the incubated cells were determined using a Quant-iT™ PicoGreen^®^ dsDNA kit (Thermo Fisher Scientific) and LabAssay ALP^®^ (FUJIFILM Wako Pure Chemical Co., Ltd.), respectively. The ALP activity was normalized to the amount of DNA in the cells.

#### 4.3.2. Measurement of Ion Concentrations and DS with Respect to Calcium Phosphate in Culture Medium

The culture media were chemically characterized to know the potential of the crystal growth and the dissolution of calcium phosphate phases. The supernatants of the culture media incubated with OCP and fetuin were collected during cell culture. The concentrations of Ca^2+^ and Pi ions in the culture medium were measured using calcium E and phosphorus C kits (FUJIFILM Wako Pure Chemical Co., Ltd.), respectively. The pH of the supernatant was measured using a pH electrode (9616S-10D; HORIBA, Ltd., Kyoto, Japan). The DS with respect to OCP, HA, and dicalcium phosphate dihydrate (DCPD) in the culture medium was calculated using Equation (2).

*DS* = (*IP*/*K_sp_*)^1/ν^, (2)

where *IP*, *K*_sp_, and *ν* are ionic activity products, the solubility product constant with respect to each calcium phosphate at 37 °C, and the number of ions in calcium phosphate, respectively. The *K*_sp_ values used for HA, OCP, and DCPD were 7.36 × 10^−60^ (mol/L)^9^ [77], 2.51 × 10^−49^ (mol/L)^8^ [78], and 2.77 × 10^−7^ (mol/L)^2^ [79], respectively. The analytical results for the Ca^2+^ and Pi concentrations and pH were used to calculate the *IP* value based on three mass balance values for Ca^2+^, Mg^2+^, and Pi ions [52,80] at an ion strength of *I* = 150 mM. In the calculations, the presence of HCO_3_^−^ and ion pairs of CaH_2_PO_4_^+^, CaHPO_4_^0^, MgHPO_4_^0^, CaHCO_3_^+^, and MgHCO_3_^+^ in the supernatants were assumed. The DS values of 1.0, <1.0, and >1.0 indicated saturation, undersaturation, and supersaturation, respectively.

#### 4.3.3. Morphological Observation and Structural Analysis of OCP After Incubations with MSCs and Fetuin

The structural and morphological information were obtained to analyze the effect of the incubation. OCP granules were collected from the inserts in the cell culture media after incubation with MSCs for 7 days and then washed three times by using ultrapure water. The washed granules were frozen at −20 °C and lyophilized for two days. The morphology of the OCP crystals in the granules after incubation was examined using FE-TEM. The chemical structure of the incubated OCP was analyzed using FTIR spectroscopy. The FTIR spectra of the specimens diluted by KBr were measured over the range of 1800–400 cm^−1^ with a resolution of 4 cm^−1^.

### 4.4. Statistical Analysis

The results are expressed as mean ± standard deviation (SD), and the differences were considered statistically significant at *p* < 0.05. Tukey–Kramer multiple comparison analysis was performed to evaluate statistical differences among the means of multiple groups. All statistical analyses were performed using Statcel 4 software (OMS Publishing Inc., Saitama, Japan). Each experiment was repeated three times independently.

## 5. Conclusions

This study demonstrated that the coexistence of OCP and the fetuin concentration close to serum level promotes the osteoblastic differentiation of MSCs. This could be taking place through the fetuin adsorption and de novo crystal formation of OCP onto the surface of the original OCP under higher DS conditions with respect to OCP and HA, the conditions of which do not suppress the intrinsic OCP capacity enhancing osteoblastic differentiation through the advancement of its hydrolysis into CDHA.

## Figures and Tables

**Table 1:** Chemical composition (Ca^2+^, Pi ion, and pH) and degree of supersaturation (DS) of culture media after the incubation of D1 cells with OCP and fetuin.

Supernatants	Periods (Day)	Ca (mM)	Pi (mM)	pH	DS at 37 °C		
					HA	OCP	DCPD
Culture medium	0	2.26	1.32	7.82	1.65 × 10^14^	5.94 × 10^4^	1.01 × 10^0^
0 mg/mL of fetuin	7	2.00	6.84	7.57	4.81 × 10^14^	7.28 × 10^5^	3.76 × 10^0^
0.2 mg/mL of fetuin	7	1.90	4.57	7.82	1.89 × 10^15^	8.74 × 10^5^	2.69 × 10^0^
0.4 mg/mL of fetuin	7	1.89	4.08	7.76	7.42 × 10^14^	4.61 × 10^5^	2.40 × 10^0^
0.8 mg/mL of fetuin	7	1.91	3.82	7.62	4.52 × 10^14^	3.31 × 10^5^	2.27 × 10^0^
0 mg/mL of fetuin + OCP	7	1.96	6.79	7.51	2.37 × 10^14^	4.81 × 10^5^	3.61 × 10^0^
0.2 mg/mL of fetuin + OCP	7	1.72	6.38	7.58	2.20 × 10^14^	3.61 × 10^5^	3.09 × 10^0^
0.4 mg/mL of fetuin + OCP	7	1.84	5.99	7.63	4.37 × 10^14^	5.35 × 10^5^	3.16 × 10^0^
0.8 mg/mL of fetuin +OCP	7	1.97	6.72	7.59	5.24 × 10^14^	7.33 × 10^5^	3.67 × 10^0^
0 mg/mL of fetuin	14	1.18	6.51	7.45	1.00 × 10^13^	4.40 × 10^4^	2.11 × 10^0^
0.2 mg/mL of fetuin	14	1.66	6.91	7.54	1.47 × 10^14^	3.09 × 10^5^	3.15 × 10^0^
0.4 mg/mL of fetuin	14	1.61	6.62	7.50	7.82 × 10^13^	2.01 × 10^5^	2.92 × 10^0^
0.8 mg/mL of fetuin	14	1.54	6.97	7.33	1.26 × 10^13^	7.41 × 10^4^	2.76 × 10^0^
0 mg/mL of fetuin + OCP	14	1.48	6.56	7.31	7.44 × 10^12^	4.93 × 10^4^	2.51 × 10^0^
0.2 mg/mL of fetuin + OCP	14	1.29	6.47	7.30	3.32 × 10^12^	2.64 × 10^4^	2.16 × 10^0^
0.4 mg/mL of fetuin + OCP	14	1.45	6.76	7.30	6.47 × 10^12^	4.60 × 10^4^	2.51 × 10^0^
0.8 mg/mL of fetuin + OCP	14	1.57	6.86	7.31	1.09 × 10^13^	6.87 × 10^4^	2.75 × 10^0^

## Data Availability

Data supporting the findings of this study are within this manuscript.
